# Comparative study of three different fixation techniques for the treatment of Neer type IIb distal clavicle fractures: A retrospective cohort study

**DOI:** 10.3389/fsurg.2023.1100720

**Published:** 2023-02-06

**Authors:** Zhi-Qing Liu, Ming-Shi Zhang, Zi-Fei Zhou, Lei Zhang, Long-Po Zheng

**Affiliations:** Department of Orthopedics, Shanghai Tenth People's Hospital, School of Medicine, Tongji University, Shanghai, China

**Keywords:** Neer type IIb distal clavicle fractures, locking plate, suture button, outcome, complications

## Abstract

**Background:**

Recently, a locking plate (LP) combined with a suture button was applied for distal clavicle Neer type IIb fractures. However, to our knowledge, there is limited information on clinical outcomes surrounding locking plates combined with a suture button in the treatment of Neer type IIb distal clavicle fractures. The aim of this study was to compare the outcomes among three different fixation techniques for the treatment of Neer type IIb distal clavicle fractures.

**Methods:**

We performed a retrospective cohort study of 53 patients with Neer type IIb distal clavicle fractures who were treated with a hook plate (HP group, 16 patients), a locking plate alone (LP group, 18 patients), or a locking plate with a suture button (LPSB group, 19 patients) in our hospital between March 2014 and August 2019. The clinical and radiological outcomes were evaluated, including union time, postoperative complications, and function of the shoulder joint.

**Results:**

The follow-up period was at least 2 years for all patients. All patients in the LPSB group achieved bone healing at the final follow-up. No significant differences were observed, including age, sex, side, time to surgery, duration of surgery, and mean follow-up period among the three groups (*p* > 0.05). The union time was shorter in the LPSB group than in the other two groups (*p* < 0.05). Postoperative complications were lower in the LPSB group than in the other two groups (*p* < 0.05). The visual analog scale score and Constant–Murley score in the LPSB group were better than those in the other groups at 3 and 6 months postoperatively (*p* < 0.05).

**Conclusion:**

Compared with HP and LP alone, LPSB yields better clinical outcomes and lower complication rates in the treatment of Neer type IIb distal clavicle fractures.

## Background

Distal clavicle fractures refer to fractures that occur in the lateral third of the clavicle, which make up approximately 10%–30% of all clavicle fractures. The management of distal clavicle fractures still represents a great clinical challenge (1, 2). The Neer classification system offers a useful framework for clinicians to assess the type of fractures. According to the Neer classification system, type IIb is an unstable fracture characterized by the obvious displacement of the fracture and additional rupture of the conoid ligament, leaving the trapezoid intact (1, 3). It is reported that unstable distal clavicle fractures have high nonunion rates when treated nonoperatively (4, 5). Thus, surgical fixation is considered for the treatment of Neer type IIb distal clavicle fractures. Surgical techniques for the treatment of distal clavicle fractures can be challenging due to comminution and large deforming forces at the fracture site (6). To date, there is no consensus regarding the best surgical approach. A range of surgical techniques have been proposed to treat distal clavicle fractures including the locking plate (LP) (7), hook plate (HP) (8), coracoclavicular (CC) fixation (9), k-wire fixation (10), and screw fixation alone (11). Each surgical approach has its own strengths and weaknesses. Moreover, many of these strategies can contribute to postoperative complications, including screw loosening or breakage, loss of reduction, and rotator cuff and acromion bone damage. Among them, locking plates and hook plates have been widely applied in the treatment of Neer type IIb fractures. Previous studies have compared the outcomes after locking plate and hook fixation for the treatment of Neer type II distal clavicle fractures, which show similar union rates (12, 13). Both surgical methods have certain complication rates and even require revision.

It was recently reported that a locking plate combined with a suture button was used for distal clavicle Neer type IIb fractures (14). However, to our knowledge, there is limited information on the clinical outcomes of the locking plates combined with a suture button in the treatment of Neer type IIb distal clavicle fractures. This study retrospectively compared the outcomes of the locking plate combined with a suture button to the hook plate and locking plate methods in the treatment of distal clavicle Neer type IIb fractures, in order to provide a better understanding and surgical options for Neer type IIb fractures.

## Methods

### Study design and patient selection

**Figure 1:**
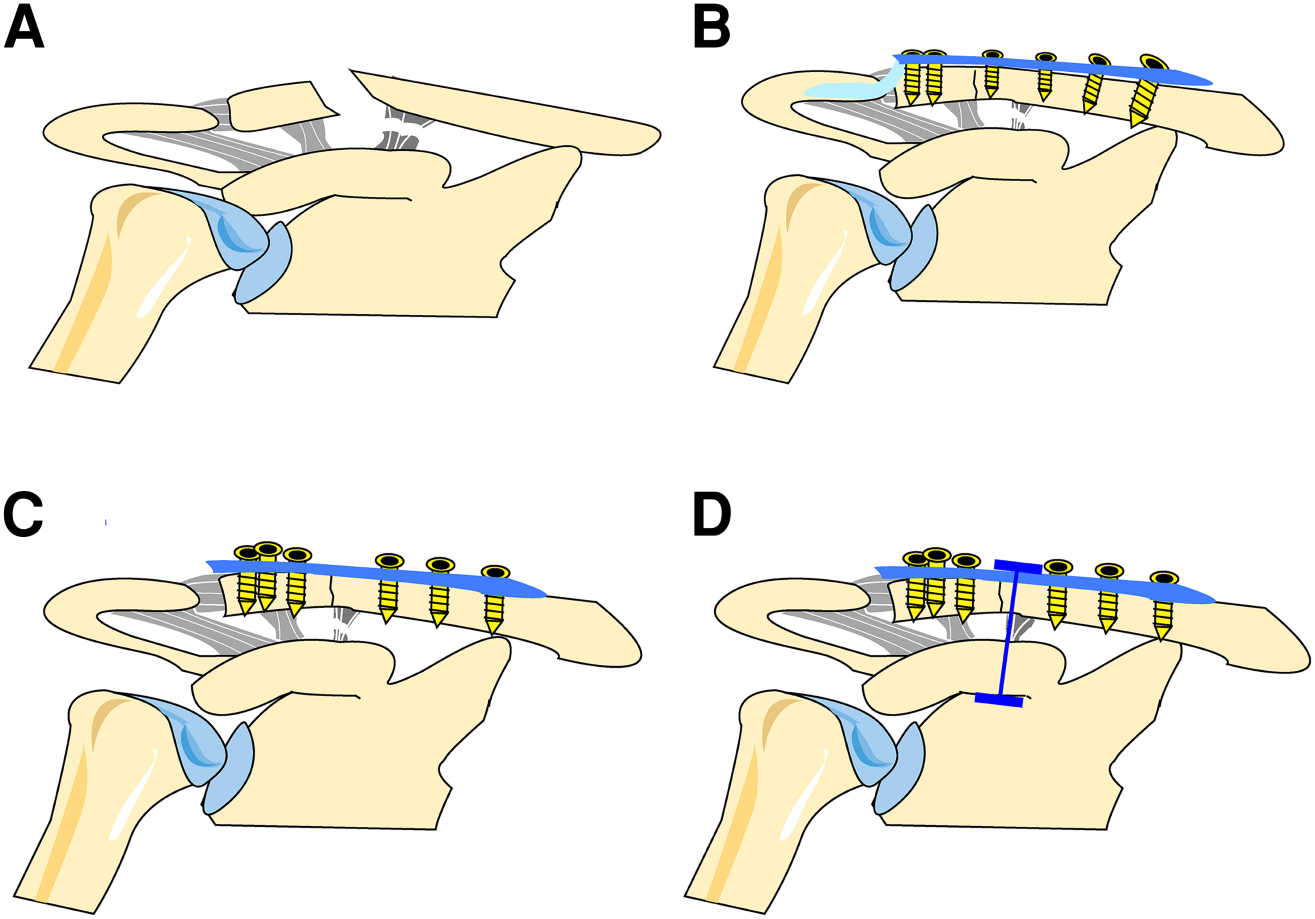
Three different fixation techniques for the treatment of Neer type IIb distal clavicle fractures. (**A**) Neer type IIb distal clavicle fractures; (**B**) HP group; (**C**) LP group; (**D**) LPSB group. HP, hook plate; LP, locking plate; LPSB, locking plate with a suture button.

**Figure 2:**
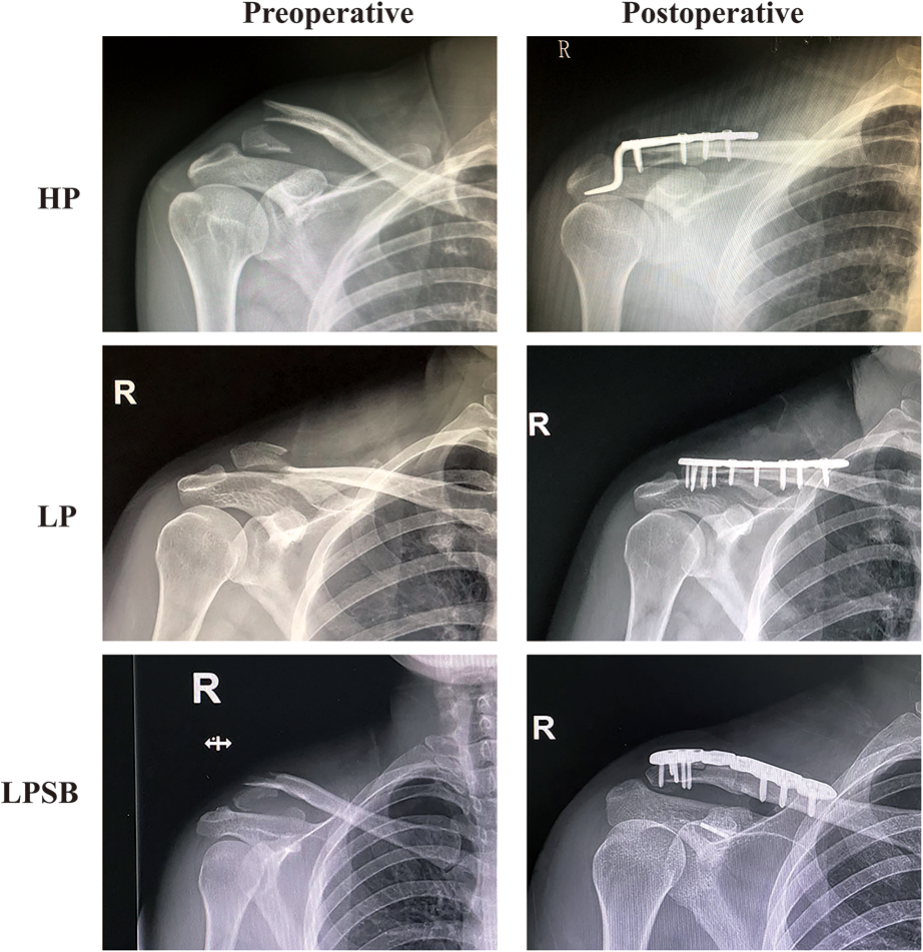
Preoperative and postoperative x-ray images in HP group, LP group, and LPSB group. HP, hook plate; LP, locking plate; LPSB, locking plate with a suture button.

Individual informed consent was waived by the Ethics Committee of the Shanghai Tenth People's Hospital due to the retrospective nature of this study. All methods were performed in accordance with the relevant guidelines and regulations, and the ethics committee at our hospital approved the study protocol. This retrospective study included 53 patients with Neer type IIb distal clavicle fractures who were admitted to our hospital between March 2014 and August 2019. The study inclusion criteria were as follows: (1) acute fractures; (2) age 18–75 years; (3) Neer type IIb distal clavicle fractures; (4) using the hook plate, locking plate alone, or locking plate with a suture button (LPSB) for internal fixation; and (5) treatment within 2 weeks after the fracture. The exclusion criteria were as follows: (1) all other types of distal clavicle fractures; (2) previous operations around the shoulder joint; and (3) pathological fractures. There were three groups in this retrospective study. Patients were treated using the hook plate (HP group, 16 patients), locking plate alone (LP group, 18 patients), or locking plate with a suture button (LPSB group, 19 patients) (Figures 1 and 2). All operations had been performed by the same orthopedic surgeon. The clinical and radiological data of the participants were collected from the electronic database of our hospital, which were reviewed and compared among the three groups.

### Surgical procedure

After a successful general anesthesia, patients were placed in the standard beach chair position. A 5-cm incision in line with the clavicle was made from the lateral clavicle to the lateral acromioclavicular margin, and the deltoid-trapezoidal fascia was incised to expose the fracture. A spinal needle was used to prevent violation of the acromioclavicular joint capsule. After cleaning the inserting soft tissue at the broken end of the fracture, the fracture was then reduced temporarily and held with two 2-mm k-wires. In the HP group, after the reduction of the fracture, the hook plate (DePuy Synthes) was utilized to fix the fracture site with locking screws. In the LP group, after the reduction of the fracture, the locking plate (DePuy Synthes) was utilized to fix the fracture site with locking screws. In the LPSB group, apart from exposing the fracture site, the coracoid process also needed to be exposed so that a suture button (TightRope; Arthrex, Naples, FL, United States) could be inserted in the base of the coracoid process. In this group, a locking plate (DePuy Synthes) with a suture button was used to fix the fracture site. The application of C-arm machine fluoroscopy confirmed that the positions of internal fixation were accurate and satisfactory.

### Postoperative management

After surgery, the shoulder was immobilized with a sling for 4 weeks, and active-movement training of the limb was prohibited. At 2 weeks postoperatively, passive shoulder exercises were performed with the assistance of the contralateral limb. At 4 weeks after surgery, the sling was removed, and the imaging findings determined whether active activities could be performed. At 6–8 weeks postoperatively, a range of joint motion exercises were encouraged. Within 3 months after surgery, extreme sports and physical labor were prohibited.

### Clinical and radiological assessment

A follow-up was performed at 4 weeks, 8 weeks, 3 months, and 6 months after surgery, and at the last follow-up. The follow-up period was at least 2 years for all patients. Fracture healing was successful when there was no tender-ness at the fracture site, and the x-ray showed that the fracture line was blurred. Bone healing was evaluated by x-ray and compared among the three groups. At 3 and 6 months postoperatively, the visual analog scale (VAS) score and Constant–Murley shoulder joint scores (CMS) were performed. During the follow-up, the occurrence of related complications was observed and counted. The complications were defined as pain, clavicle shaft fractures and dislocation of the hook, acromial erosion, acromioclavicular joint dislocation, and distal screw extraction.

### Statistical analysis

Statistical analysis was performed using SPSS version 22.0 statistical software. According to the normality and homogeneity of the variance of the data, continuous variables were compared by ANOVA or Kruskal–Wallis test for multiple group comparison and Student's *t*-test or Mann–Whitney test for the individual group comparison. Categorical data were analyzed using the chi-square test. Statistical significance was defined as *p* < 0.05.

## Results

### General results

The patient characteristics of the study population at baseline before surgery are shown in Table 1. A total of 53 patients with Neer type IIb distal clavicle fractures were involved. No differences were observed regarding patient characteristics including age, sex, side, and follow-up among the three groups (*p* > 0.05).

**Table 1:** Baseline characteristics of the three groups.

Characteristics	HP (*n* = 16)	LP (*n* = 18)	LPSB (*n* = 19)	*p* value
Age (years)	44.3 ± 11.1	39.7 ± 9.1	41.9 ± 11.8	0.455
Gender (male/female)	7/9	8/10	8/11	0.989
Side (left/right)	9/7	6/12	7/12	0.350
Time to surgery (days)	3.3 ± 1.0	2.8 ± 1.1	3.0 ± 1.0	0.455
Duration of surgery (min)	31.9 ± 4.7	32.4 ± 4.6	47.7 ± 4.0	<0.05*
Follow-up (months)	25 (24–27)	26 (24–28.25)	26 (24–27)	0.758

HP, hook plate; LP, locking plate; LPSB, locking plate with a suture button. *p-value less than 0.05.

### Outcomes

Union was achieved in all patients finally. The VAS score and CMS in the LPSB group were better than those in the other two groups (*p* < 0.05), which are shown in Table 2.

**Table 2:** Postoperative complications and outcomes of the three groups.

	HP (*n* = 16)	LP (*n* = 18)	LPSB (*n* = 19)	*p* value
Complications, *n* (%)	5 (31.3)	2 (11.1)	0 (0)	<0.05*
Union time (weeks)	14.0 ± 1.67	13.8 ± 1.8	11.1 ± 1.9	<0.01**
VAS-3	4 (3.3–4)	3 (2–4.25)	3 (2–3)	<0.01**
CMS-3	70.6 ± 6.0	83.9 ± 4.5	91.4 ± 4.9	<0.01**
VAS-6	2.5 (2–3)	2 (1–3)	1 (1–2)	<0.01**
CMS-6	88.1 ± 4.0	89.1 ± 2.8	95.6 ± 2.5	<0.01**

HP, hook plate; LP, locking plate; LPSB, locking plate with a suture button; VAS, visual analog scale score; CMS, Constant–Murley score. *p-value less than 0.05, **p-value less than 0.01.

### Complications

**Figure 3:**
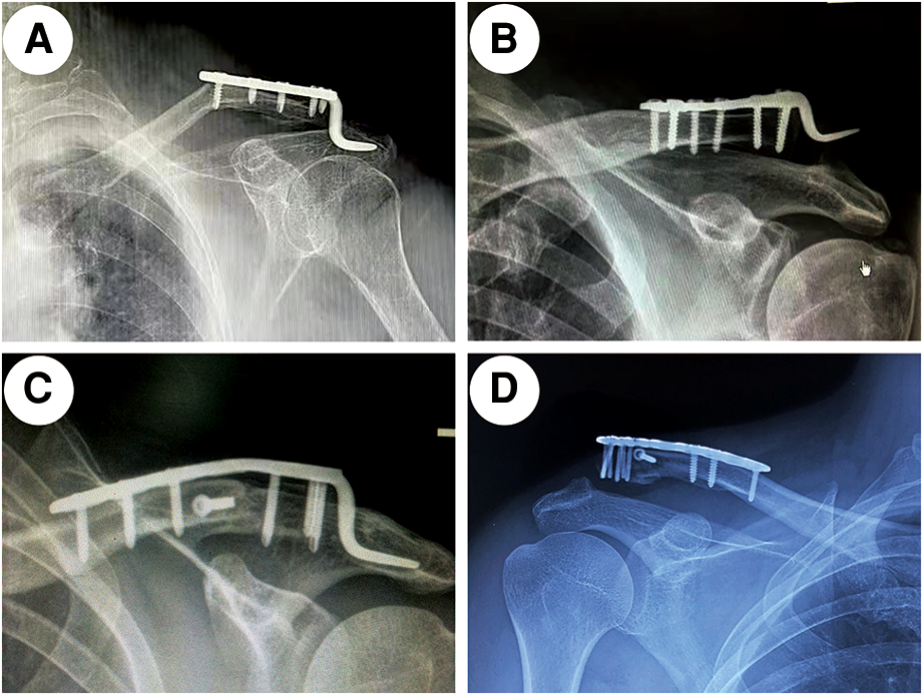
The postoperative complications in the HP group and LP group. (**A**) Clavicle shaft fracture, (**B**) dislocation of hook, (**C**) acromial erosion, and (**D**) acromioclavicular joint dislocation. HP, hook plate; LP, locking plate.

In terms of postoperative complications, in the HP group, clavicle shaft fractures and dislocation of the hook occurred in one patient each (Figure 3). The patient with clavicle shaft fractures underwent revision surgery using the longer clavicular hook plate, and the patient with dislocation was treated with LPSB. These two patients underwent complete revision and achieved union finally. In addition, two patients who had symptoms of pain in the final follow-up developed acromion erosions. In the LP group, acromioclavicular joint dislocation (Rockwood Grade-III) and distal screw extraction occurred in two patients and one patient, respectively (Figure 3). The patients with acromioclavicular joint dislocation were immobilized with a sling for 6 weeks. Revision surgery was performed on the patient with distal screw extraction. In the LPSB group, no dislocation and no screw pullout were observed. Postoperative complications were lower in the LPSB group than in the other two groups (*p* < 0.05).

## Discussion

Distal clavicle fractures have a great impact on shoulder joint function. Among them, Neer type IIb fractures have a high fracture nonunion rate due to severe fracture displacement, which remains a huge challenge in clinical treatment (15). At present, surgical treatment is recommended for type IIb fractures, but the surgical options are still controversial (16). Each method has its strengths and weaknesses. The aim of this study was to compare the outcomes among three different fixation techniques for the treatment of Neer type IIb distal clavicle fractures.

### Complications in the HP and LP groups

The complications of unstable distal clavicular fractures, such as nonunion or delayed union, are likely to occur due to their specific biomechanical configuration (17). Although the hook plate has been demonstrated as an efficient way to treat distal clavicular fractures in past few decades, implant removal is necessary. In addition, many complications occurred frequently (18). In the present study, our results are consistent with those of a previous study (12), and clavicle shaft fractures, dislocation of the hook, and acromial erosion occurred. In agreement with a previous study (13), our results indicated that compared with the HP, the LP was associated with fewer complications. Although the LP is an acceptable method of treating distal clavicle fractures, it does not solve the problem of coracoclavicular ligament rupture. In the present study, two patients had acromioclavicular joint dislocation and one patient had distal fragment screw pullout after treatment with the LP. We believe that this may be related to the fact that the coracoclavicular ligament was not reconstructed, and the stress was concentrated in the distal end fracture fragments and acromioclavicular joint. The coracoclavicular ligament is an important component of the body, is one of the important structures that constitutes the static stability of the joint, and it has the function of resisting a large displacement moment. Therefore, an LP with supplementary coracoclavicular fixation has gradually become a trend.

### Outcomes among three different fixation techniques

In the present study, our results indicated that compared with the HP, the LP was associated with lower pain and higher shoulder joint function scores in the short follow-up. With the extension of follow-up time, no significant differences were observed between the two groups in terms of pain and higher shoulder joint function scores. There was also no significant difference in terms of healing time between the two groups. However, compared with the HP, the LP was associated with fewer complications. Thus, the LP was superior to the HP in the treatment of distal clavicle fractures. As mentioned above, the LP has also certain complications. The LP with a supplementary coracoclavicular fixation in the treatment of distal clavicle fractures should be considered. Clinically, there is no consensus standard for coracoclavicular ligament reconstruction. It is reported that the application of the suture button for augmentation of the coracoclavicular ligament has a good therapeutic effect on the fixation of distal clavicle fractures (19). In this study, the locking plate with a suture button group was compared with the other two groups. Although the procedure of the locking plate with a suture button is relatively complicated, compared with the HP and the LP, the LPSB in the treatment of distal clavicle fractures has the advantages of early postoperative functional recovery, a higher shoulder joint score, and fewer complications (Table 2). The suture button reconstructs the coracoclavicular ligament and resists the upward displacement force of the clavicle, and the LP mainly eliminates the anterior and posterior displacement force of the clavicle, so it has achieved good results. In the present study, the operation time in the LPSB group was significantly higher than that in the other groups (*p* < 0.05). This was mainly because the coracoid process was exposed and a suture button was added. Compared with the LP group, the LPSB group was associated with higher surgery costs. However, there was no significant difference between the LPSB and HP groups. It was mainly because implant removal was needed in the HP group.

### Limitations

The present study has some limitations. First, it was a retrospective study, which may have selection bias. In addition, a relatively low number of individuals were involved in the study. Thus, randomized controlled trials need to be carried out, and it is necessary to further enlarge the sample size to validate the findings of the present study.

## Conclusion

In conclusion, the locking plate combined with a suture button is an excellent surgical choice for the treatment of Neer type IIb distal clavicle fractures, which has the advantage of fewer complications and can make up for the deficiency of a single locking plate treatment.

## Data Availability

The raw data supporting the conclusions of this article will be made available by the authors, without undue reservation.
